# Diagnosis and management of tuberculosis infection in inclusion health populations in London

**DOI:** 10.1186/s12879-024-09132-3

**Published:** 2024-02-23

**Authors:** Adam Gray, Julian Surey, Martha Veitch, Dee Menezes, John Gibbons, Mark Leonard, Binta Sultan, Hanif Esmail, Al Story

**Affiliations:** 1 University College London Hospitals, London, UK; 2 Hospital for Tropical Diseases, University College London Hospitals, London, UK; 3 Institute for Global Health, University College London, London, UK; 4 Universidad Autonoma de Madrid, Madrid, Spain; 5 Public Health Data Science, Institute of Health Informatics, University College London, London, UK; 6 TB Service, Whittington Hospital, London, UK; 7 Collaborative Centre for Inclusion Health, University College London, London, UK

**Keywords:** TB infection, Inclusion health, Homelessness

## Abstract

**Background:**

Tuberculosis in the UK is more prevalent in people with social risk factors– e.g. previous incarceration, homelessness - and in migrants from TB endemic countries. The management of TB infection is part of TB elimination strategies, but is challenging to provide to socially excluded groups and the evidence base for effective interventions is small.

**Methods:**

We evaluated a TB infection screening and treatment programme provided by a peer-led service (Find&Treat) working in inclusion health settings (e.g. homeless hostels) in London. IGRA (interferon-gamma release assay) testing and TB infection treatment were offered to eligible adults using a community-based model. The primary outcome was successful progression through the cascade of care. We also evaluated socio-demographic characteristics associated with a positive IGRA.

**Results:**

42/312 (13.5%) participants had a positive IGRA and no one had evidence of active TB. 35/42 completed a medical evaluation; 22 started treatment, and 17 completed treatment. Having a positive IGRA was associated with previous incarceration and being born outside of the UK.

**Discussion:**

Provision of TB infection diagnosis and management to this socially excluded population has several challenges including maintaining people in care and drug-drug interactions. Peer-support workers provided this service safely and effectively with appropriate support. Further work to generate data to inform risks and benefits of treatment for TB infection in this group is needed to facilitate joint decision making.

**Supplementary Information:**

The online version contains supplementary material available at 10.1186/s12879-024-09132-3.

## Background

The epidemiology of tuberculosis (TB) in low burden settings like the United Kingdom (UK) demonstrates significant heterogeneity– people in the most deprived decile have a fivefold risk of infection compared to those in the least deprived decile [1] and have worse clinical outcomes [2]. The emerging umbrella term “inclusion health” describes a heterogenous population defined by systematically reduced access to healthcare [3, 4] and this group typically include those with a “social risk factor” (SRF) for TB– e.g. illicit drug use, homelessness, excess alcohol use, previous incarceration.

In 2019 in the UK, 13.9% of people notified with TB had a SRF, and this group had worse TB outcomes as compared to those with no SRF– a higher proportion were lost-to-follow-up (6.2% vs. 2.7%), a lower proportion completed treatment (77.7% vs. 87.7%) and a higher proportion died (7.1% vs. 3.5%) [5]. For countries targeting TB elimination, public health strategies should effectively reach these groups [6].

A pillar of TB reduction initiatives is management of TB infection (TBI) [7]. In the UK, TBI screening is routinely conducted for some populations at increased risk of progression to active TB, including those due to receive TNF-alpha inhibitors and migrants from TB endemic countries [8]. Rates of TBI in inclusion health populations are high globally [9–14] and within the UK being part of the inclusion health population is a risk factor for TBI independent from country of origin [8, 15]. Although UK national guidance recommend screening these groups for TBI [16], this is not routinely done due to several factors including lack of funding [17, 18].

When compared to the general population, treatment completion rates for TBI in inclusion health populations are lower [19] and there are specific issues around toxicity monitoring and drug-drug interactions (e.g. rifampicin-opiate interaction) [20]. Potential approaches have included incentives, peer support, and alternative treatment regimens [21] such as 3HP (three months of weekly rifapentine and isoniazid), which may be more amenable to directly observed treatment [22, 23].

We sought to evaluate a community-based model of care for the diagnosis and treatment of TBI for an inclusion health population in London, with a primary outcome of successful treatment completion.

## Methods

### Population and setting

From October 2021 to August 2022, individuals screened during routine work by the Find&Treat mobile teams were additionally offered TBI testing if they met NICE (National Institute for Health and Care Excellence) criteria: (a) aged 18–65 and (b) homeless or previously using illicit drugs or previously incarcerated or currently drinking excess alcohol [16].

Screening was conducted by Find&Treat, a peer-led, NHS-commissioned service that has provided health-screening to inclusion health populations around London for 20 years. This is conducted on two mobile health units - one with x-ray capability (mobile X-ray unit [MXU]) and one without (mobile health unit [MHU]) - which visit homeless hostels, drug and alcohol services, and day centres. Individuals are offered chest x-ray (CXR) screening for active TB and point-of-care testing for hepatitis C, hepatitis B, HIV, and syphilis. Homeless hostels in the UK are mostly run by private providers who must maintain adequate living standards; residents have single-occupancy rooms.

### Procedures

Individuals were offered screening for TBI with an interferon-gamma release assay (IGRA) test (QuantiFERON®-TB Gold In-Tube) conducted at University College London Hospitals NHS Foundation Trust. Basic socio-demographic data was collected at time of screening with testing conducted by a peer-worker, nurse, or physician. All participants with a positive IGRA underwent a medical evaluation by an experienced TB clinician, including an assessment of co-morbidities, the probability and hazard of progression from infection to active disease, and potential for treatment-related harms. Participants with a positive IGRA tested on the MHU (without x-ray capacity) had a CXR arranged at their local clinic with attendance supported by a peer-worker. All participants with CXR abnormalities or symptoms suggestive of active TB submitted sputum for TB smear and culture. Participants diagnosed with TBI - defined as having a positive IGRA, but no clinical or radiographic features for active TB disease– were offered treatment. Participants also diagnosed with active hepatitis C infection were referred for treatment prior to considering TBI treatment.

Treatment options were limited to those routinely use in the UK: 3 h (three months of daily rifampicin and isoniazid), 4R (four months of daily rifampicin), or 6 H (six months of daily isoniazid). Participants using illicit opiates were eligible for rifampicin-containing regimens if they were receiving a stable dose of opiate-substitution therapy (OST) but were counselled regarding the interaction and had an appointment with their OST prescriber arranged for day three of TBI treatment. Participants who drank hazardous amounts of alcohol were not offered isoniazid-containing regimens given increased risk of hepatotoxicity and the anticipated limitations on regular monitoring [24]. A full description of the treatment decision algorithm is available (Supplementary material).

Initial and follow-up assessments were conducted in the community by a TB clinician, typically the same place as diagnosis– e.g. homeless hostel. The individuals being treated for TBI were followed up at two weeks for drug safety monitoring (e.g. liver function tests), and then monthly to monitor adherence (pill count or self-report), review for adverse events, and deliver the next month’s supply of medications. If a low proportion of medication was taken (less than 80% in any month) this was reviewed and a plan to continue, stop, or re-start jointly made with the individual.

### Outcomes

The primary outcome was the proportion of those with a positive IGRA who successfully completed treatment– defined as at least 80% of doses taken. Secondary outcomes included proportion of people screened who had a positive IGRA and presence of any adverse events.

### Statistical analysis

We aimed to identify 50 people with TBI with the expectation this would generate sufficient information on progression through the cascade of care. Using previous estimates of TBI prevalence (16%) [15] we aimed to screen 312 individuals. This sample size would also power an estimate of the prevalence of TBI at 16% with a 4% margin of error and 95% confidence intervals (95% CI).

Participant socio-demographic characteristics and progression through the cascade of care are reported using percentages, and prevalence of a positive IGRA shown as a number-needed-to-screen. A univariate analysis using odds ratios (OR) assessed associations between a positive IGRA and selected characteristics including age, gender, region of birth (UK vs. non-UK, non-TB endemic vs. TB-endemic (TB prevalence > 100/100,000 in 2021) [2]), and previous incarceration. The sampling framework was non-random, with both health units purposively focusing on settings known to have high rates of alcohol and illicit drug use; we felt it inappropriate to assess these as dependent variables. All analyses were conducted using R (Version 4.2.2, R Foundation for Statistical Computing, Vienna) [25].

### PPI (patient and public involvement)

Peer support workers (PSWs), with experience of homelessness and/or drug use, helped write the protocol and develop the model of care.

## Results

### Participant characteristics

324 individuals had an IGRA performed. The majority were male (263, 81%) with a median age of 46 years old (Table 1). Of those with documented responses, over half were drinking alcohol daily and over half had previously or were currently using illicit drugs (crack cocaine or heroin), although prevalence of substance misuse was much higher in UK born individuals. 145/324 (44.8%) participants were born outside of the UK, 111/145 (76.6%) of whom were from a country with a low TB incidence. The median time in the UK at time of testing was 17 years (range 0–61 years). 179/324 (55%) participants were tested on the MXU and 145/324 (45%) on the MHU; the demographics of participants were similar on both units, although rates of HCV antibody were higher in those screened on the MHU– this was anticipated as the MHU prioritised hostels with higher prevalence of drug use (Supplementary Table S1).

Only limited information on people who did not participate in TBI screening was available − 23/168 (13.7%) eligible participants on the MHU did not have IGRA testing due to refusal or lack of venous access. On the busier MXU, 243/422 (58%) of potentially eligible participants did not have IGRA testing predominantly due to workflow constraints: the process of phlebotomy for IGRA took longer than that for CXR and there was only one phlebotomist. The questionnaires used on the MXU and MHU were different (e.g. alcohol use was not asked about on the MXU), resulting in missing data as this was not collected retrospectively.

**Table 1:** Socio-demographic characteristics of study participants by region of birth

Characteristic	Born in UK (*n* = 178)	Born in non-TB endemic country (exc. UK) (*n* = 112)	Born in TB-endemic country (*n* = 34)	Total (*N* = 324)
Age (years)– median (IQR)	48 (39–55)	44 (36–51)	46 (34–55)	46 (37–54)
Male gender– no. (%)	140 (80.9)	91 (81.2)	27 (79.4)	263 (81.2)
**Illicit drug use– no. (%)**				
Current	104 (58.4)	31 (27.7)	6 (17.6)	141 (43.5)
Past	36 (20.2)	13 (11.6)	1 (2.9)	50 (15.4)
Never	22 (12.4)	51 (45.5)	19 (55.9)	92 (28.4)
Unknown	16 (9.0)	17 (15.3)	8 (23.5)	41 (12.7)
HCV Ab reactive– no. (%)	90 (50.6)	28 (25.0)	3 (8.8)	121 (37.3)
**Alcohol use– no. (%)**				
Current daily drinking	73 (41.0)	29 (25.9)	9 (26.5)	111 (34.3)
Not drinking, or less than daily	36 (20.3)	41 (36.6)	10 (29.4)	87 (26.9)
Not documented	69 (38.8)	42 (37.5)	15 (44.1)	126 (38.9)
**Previous incarceration– no. (%) (%)**				
Yes	75 (42.1)	28 (25.0)	8 (23.5)	111 (34.3)
No	88 (49.4)	67 (59.8)	23 (67.6)	178 (54.9)
Unknown	15 (8.4)	17 (15.2)	3 (8.8)	35 (10.8)
**Site Type– no. (%)**				
Homeless hostel	100 (56.2)	76 (67.9)	26 (76.5)	202 (62.3)
Drug service	43 (24.2)	13 (11.6)	4 (11.8)	60 (18.5)
Day centre	35 (19.7)	22 (20.5)	4 (11.8)	62 (19.1)

### Primary outcome: completion of care cascade

**Fig. 1:**
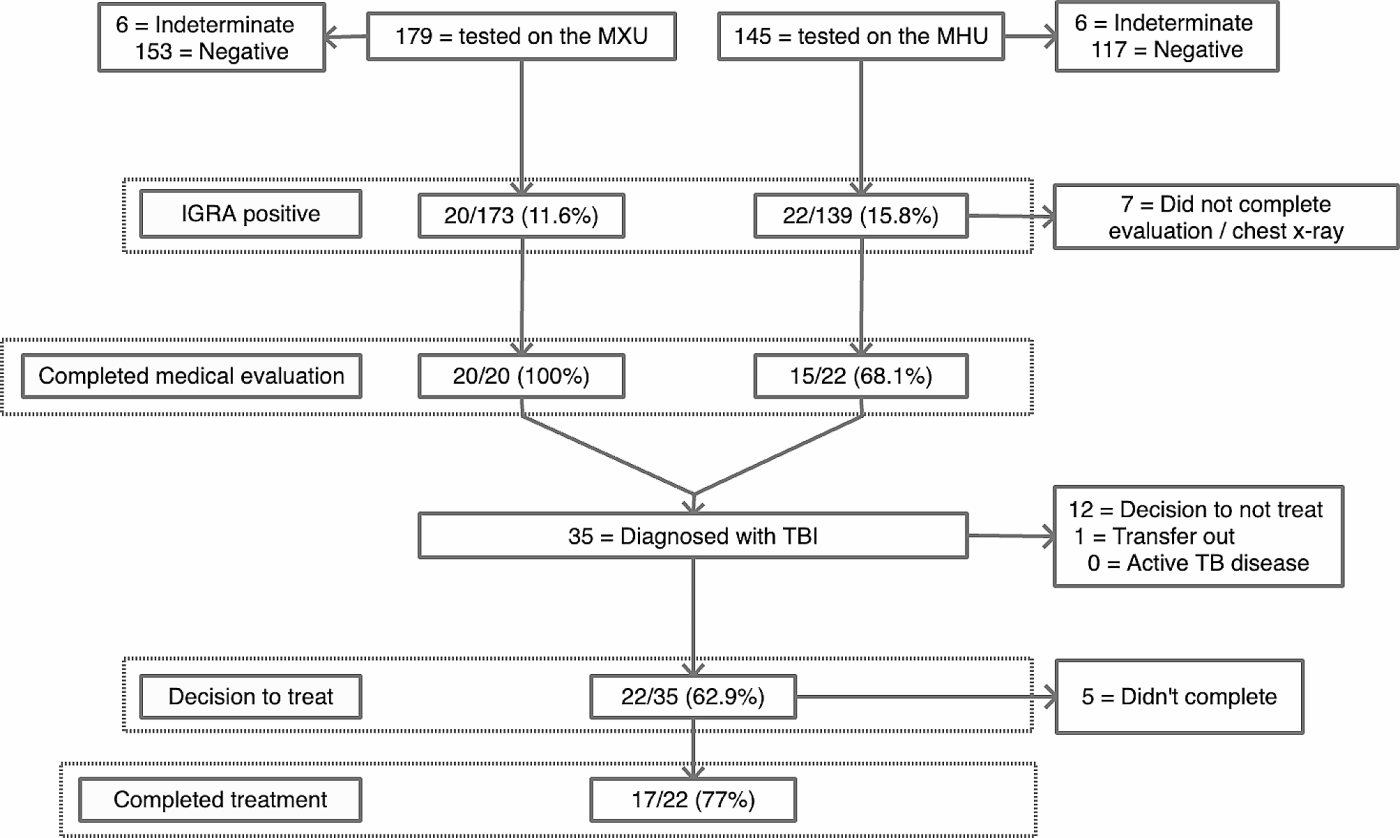
Diagnostic and treatment cascade of care for TBI

42/312 (13.5%) IGRAs with valid results were positive with a number-needed-to-screen (NNS) of 7. 12/324 (3.7%) were indeterminate and attempts were not made to repeat these unless the participant was considered at particularly elevated risk of progression to active TB disease. 35/42 participants with a positive IGRA completed a medical evaluation including a CXR (Fig. 1). 7/42 did not, all of whom had been screened on the MHU (without CXR capacity) and did not attend their local clinic for CXR despite support from peer support workers. One individual with a normal CXR had symptoms potentially in keeping with active TB and had sputum testing conducted. No-one was diagnosed with active TB.

No participants had absolute contra-indications to treatment options despite substantial drug and alcohol use, medical co-morbidity (23/42 had at least one medical co-morbidity), and mental health problems (15/42). 12/35 (34%) of those with a complete medical assessment declined treatment with reasons including competing priorities (e.g. currently sleeping on the street), participant concerns about impact from rifampicin on methadone dose stability, and long duration of the rifampicin-free regimen. It was not possible to ascertain the probable timing of TB infection for most participants, and other than for two immunocompromised individuals, the potential individual benefit of treatment was generally felt to be low. A joint decision to start treatment was made with 22/35 (62.9%), with one person subsequently leaving the country before starting. 6/22 started on TBI treatment were using opiates (prescribed or illicit); 4/6 of whom were treated with a rifampicin-containing regimen.

17/22 (77%) of those who started treatment successfully completed more than 80% of prescribed doses. Participants prescribed shorter regimens were more likely to complete treatment: 7/7 (100%) 3 h, 10/13 (77%) 4R, and 0/2 (0%) 6 H. Hazardous alcohol use and ‘competing priorities’ were the key underlying reason for non-completion. Regular communication through follow-up was anticipated to be challenging and only 21/42 (50%) had a mobile phone which they answered, although with consent we communicated via the participant’s key worker at their hostel accommodation with good success and no participants started on treatment were lost-to-follow-up.

### Secondary outcomes

#### Safety outcomes

One participant developed opiate withdrawal symptoms three days after starting rifampicin; their OST was subsequently adjusted but they chose not to restart TBI treatment. Baseline liver function tests in 8/42 (19%) were abnormal (alanine transaminase (ALT) 1–3*upper limit of normal) - none of these individuals had active viral hepatitis, but all were hazardous alcohol users and were not offered an isoniazid-based regimen. One individual had an ALT rise from baseline during treatment which self-resolved without treatment interruption. No other adverse events were identified.

#### Prevalence of TBI

The prevalence of TBI was 13.5% (95% CI 11.6–15.4) and we explored whether there was population heterogeneity using select pre-specified variables (Table 2). The percentage of people with TBI who were UK-born (9.7%, 95% CI 5.3–14.1) was lower than for people born elsewhere in non-TB endemic countries (14.3%, 95% CI 7.6–21.0) and in TB-endemic countries (31.2%, 95% CI 15.2–47.3), but much higher than estimates for the general UK population (1.6%) [26]. TBI was associated with previous incarceration - although rates of incarceration were higher in those born in the UK (47%) compared to Europe (35%) and elsewhere (22%).

**Table 2:** Risk factors for TBI by percentage and univariate regression

Characteristic	IGRA Positivity 42/312 (13.5%)	Odds Ratio (95% CI)	p-value (chi sq.)
**Age**			
18–30	1/33 (3.0)	1.00	
31–40	6/67 (9.0)	2.81 (0.44–75.03)	0.27
41–50	18/96 (18.8)	6.48 (1.25–160.04)	0.03
51–65	17/116 (14.7)	4.83 (0.93–119.54)	0.07
**Gender**			
Male	35/251 (13.9)	1.00	
Female	7/61 (11.5)	0.81 (0.31–1.84)	0.61
**Region of Birth**			
Born in UK	17/175 (9.7)	1.00	
Born in non-TB endemic country (exc. UK)	15/105 (14.3)	1.55 (0.73–3.27)	0.24
Born in TB endemic country	10/32 (31.2)	4.20 (1.65–10.34)	< 0.001
Previous incarceration*			
No	14/173 (8.1)	1.00	
Yes	26/110 (23.6)	3.48 (1.74–7.23)	< 0.001

*Missing incarceration data for 29 individuals

## Discussion

This evaluation found that a community-based service offering TBI diagnosis and treatment to an inclusion health population found a high prevalence of TBI and safely and effectively provided treatment to a reasonable proportion. However, there were substantial challenges throughout the cascade of care. Despite a highly experienced multi-disciplinary team, a third of those screened on the mobile unit without CXR capacity did not complete a medical assessment. A third of those who did complete assessment declined treatment, broadly because treatment did not feel relevant or important to the individual at that time. This resulted in 17/42 (40.5%) of those with a positive IGRA completing treatment for TBI. Much of this service was provided by peer-support workers, and this evaluation supports their capacity in this regard in the context of a robust, well supported system. The prevalence of TBI in our cohort was heterogenous, with individuals born in TB endemic countries, or with previous incarceration being at increased risk, risks well established in the literature. Future studies are required to unpick how co-linear risks (e.g. incarceration, illicit drug use, homelessness) relate to each other. TBI testing in this population NNS of 7 to identify one person with TBI. Further work is needed to better understand the number-needed-to-harm (NNH) and NNS to prevent one person from developing active TB. Limiting screening to people with the greatest probability of TBI (e.g. previous incarceration or non-UK born) would clearly reduce the NNS but would introduce further health inequity into an already marginalised population.

TBI treatment using a community-based approach, designed purposefully for inclusion health populations has been effective previously in the USA [27] and has been piloted on a small scale with sex-workers within the UK [28]. A recent review with recommendations found that holistic approaches, including support with housing and food, enhanced treatment adherence in people experiencing homelessness, and that testing and treatment for TBI prevented progression to TB disease [29].

This evaluation was of a service conducted by Find & Treat, a well-established, holistic healthcare screening and support service that has been shown to be effective and cost-effective for active TB screening [30] and for hepatitis C [31]. The use of peer-support workers and combination of screening for TBI alongside other issues– including substance misuse– would likely result in this service being more cost-effective when compared to a novel vertical programme, although further work is required to establish the utility of TBI screening in this specific population. Although aspects of TBI management are complex (e.g. risk evaluation of treatment), much of the process is amenable to being conducted using a protocol, which might enhance the role of peer-support workers.

Rifampicin-opiate interactions were an important part of decision making both for the clinician and the person being treated for TBI and brought additional risk to treatment for a population that were often difficult to maintain contact with. Close co-ordination with drug and alcohol services and client education was required, but novel regimens with less problematic drugs would clearly be of benefit to this population - weekly isoniazid and rifapentine (3HP) has been effectively used in homeless populations [32, 33], and could be provided using pharmacy DOT for people receiving OST. Whilst hostel keyworkers are in an advantageous position to encourage adherence for those residing in homeless hostels, they are not currently able to manage medications or provide DOT– training and support might use this opportunity to upskill an important cadre.

This new TBI service has several strengths including the pragmatic application of national guidance within an already functioning screening service, utilising peer-support workers to optimise uptake of testing and treatment. The key weakness of this service is its generalisability to other settings with different TB epidemiology or different resource capacity. Additionally, the rates of progression to active disease in the inclusion health population is unknown and so the impact from this intervention on individual or community risk is uncertain. In addition, this was an NHS service aiming to offer screening to highly marginalised groups, therefore this convenience sample may not be representative of the wider homeless population.

## Conclusion

This pilot of community-based diagnosis and management of TBI in an inclusion health population required substantial resource and multi-disciplinary relationships but was effective and safe. Whilst cost-effectiveness assessments are often key to generating funding, providing equitable care to marginalised populations to achieve community-wide elimination of TB should provide a strong enough mandate.

### Electronic supplementary material

Below is the link to the electronic supplementary material.

Supplementary Material 1

## Data Availability

The datasets used and analysed during the current study are available from the corresponding author on reasonable request.

## Abbreviations

3HP: 3 (months) isoniazid and rifapentine (12 weekly doses)
3 h: 3 (months) isoniazid and rifampicin
4R: 4 (months) rifampicin
6 H: 6 (months) isoniazid
ALT: alanine transaminase
DOT: Directly observed therapy
CXR: Chest x-ray
HIV: Human immunodeficiency virus
IGRA: Interferon-gamma release assay
MHU: Mobile health unit
MXU: Mobile x-ray unit
NHS: National health service
NICE: National Institute for Health and Care Excellence
NNS: Number needed to screen
OST: Opiate substitute treatment
PSW: Peer-support worker
SRF: Social risk factor
TB: Tuberculosis
TBI: TB infection
TNF: Tumour necrosis factor
