# Supplementary material for: Diagnosis and management of tuberculosis infection in inclusion health populations in London
Source: BMC Infect Dis. 2024 Feb 23;24:252. doi: 10.1186/s12879-024-09132-3 (PMC10893593; doi:10.1186/s12879-024-09132-3)
Supplement: Supplementary file 1 — Supplementary Material 1 [file 12879_2024_9132_MOESM1_ESM.docx]

# Diagnosis and management of latent tuberculosis infection in inclusion health populations in London:

# Supplementary Material

### Supplementary Material: LTBI Treatment Decision Tool

Step 1: Treatment appropriateness

An assessment of risk of progression to TB disease is made by the clinician using timing of history of TB exposure or immigration from a high burden setting, past medical history, and quantitative value of IGRA. This was presented to the patient and contextualised with an assessment of how achievable taking regular medications for 3-6 months would be given potential for toxicity and monitoring.

Step 2: Isoniazid suitability

Absolute contra-indications included: daily alcohol use, serious drug-drug interactions

Isoniazid may also be considered contra-indicated based on a risk/benefit assessment considering factors including age, alcohol use, current or previous liver disease, and baseline liver function tests.

Step 3: Rifampicin suitability

Absolute contra-indications included: serious drug-drug interactions, regular illicit opiate use in someone not receiving opiate substitution therapy

Patients receiving opiate substitution therapy had to agree to close monitoring with their drug and alcohol service, and consent to up titration. Patients using illicit opiate very occasionally were able to receive rifampicin as the risk of opiate withdrawal would be very low.

In practice, it was found that few people with regular illicit opiate use were in a position to take a six month treatment regimen. For those able to take a rifampicin containing regimen, it was often felt the risk of isoniazid toxicity (elevated for many of this population) outweighed the benefit of a slightly shorter regimen and were therefore offered 4R.

Isoniazid contra-indicated

Yes

No

Rifampicin contra-indicated

Rifampicin contra-indicated

No

Yes

No

Yes

3HR

6H

4R

Nil

Participants form whom no suitable treatment option was considered appropriate had the risks of progression explained to them and communicated to their primary care team.

### Supplementary Table S1: Patient characteristics stratified by testing location

| Characteristic | Location | |
| --- | --- | --- |
|  | **MXU (n=179)** | **MHU (n=145)** |
| Age (years) – Median (IQR) | 45 (35-54) | 48 (41-55) |
| Male gender – no. (%) | 141 (78.8) | 122 (84.1) |
| Region of birth – no. (%) |  |  |
| UK | 85 (47.5) | 93 (64.1) |
| Non-TB endemic (exc. UK) | 69 (38.5) | 43 (29.7) |
| TB endemic country | 25 (14.0) | 9 (6.2) |
| HCV Ab – no. (%) |  |  |
| Reactive | 19 (10.6) | 102 (70.3) |
| Unreactive | 159 (88.8) | 43 (29.7) |
| Unknown | 1 (0.6) |  |
| Drug use – no. (%) |  |  |
| Current | 64 (35.8) | 77 (53.1) |
| Past | 14 (7.8) | 36 (24.8) |
| Never | 64 (35.8) | 28 (19.3) |
| Unknown | 37 (20.7) | 4 (2.8) |
| Alcohol use – no. (%) |  |  |
| Current daily drinking | 32 (17.9) | 79 (54.5) |
| Not drinking, or less than daily | 34 (19.0) | 53 (36.6) |
| Unknown | 113 (63.1) | 13 (9.0) |
| Previous incarceration – no. (%) |  |  |
| Yes | 55 (30.7) | 56 (38.6) |
| No | 114 (63.7) | 64 (44.1) |
| Unknown | 10 (5.6) | 25 (17.2) |
| Site Type – no. (%) |  |  |
| Homeless hostel | 91 (50.8) | 111 (76.6) |
| Drug service | 41 (22.9) | 19 (13.1) |
| Day centre | 47 (26.3) | 15 (10.3) |
